# Understanding the dynamics of SARS-CoV-2 variants of concern in Ontario, Canada: a modeling study

**DOI:** 10.1038/s41598-022-06159-x

**Published:** 2022-02-08

**Authors:** Anita T. Layton, Mehrshad Sadria

**Affiliations:** 1 Department of Applied Mathematics, University of Waterloo, Waterloo, ON Canada; 2 Department of Biology, Cheriton School of Computer Science, School of Pharmacy, University of Waterloo, Waterloo, ON Canada

**Keywords:** Computational biology and bioinformatics, Diseases

## Abstract

A year after the initial wild-type Severe Acute Respiratory Syndrome Coronavirus 2 (SARS-CoV-2) strains began their devastation of the world, they were supplanted by new variants of concern (VOC). In Ontario, Canada, the wild type was overtaken first by the Alpha/B1.1.17 variant, and then by the Delta/B.1.617 variant. The principal objective of the present study is to develop and apply a much expanded Susceptible-Infection-Recovered-type model to better understand the spread of multiple VOC, and assess the effectiveness of vaccination and non-pharmaceutical interventions (NPI). The model represents competition among VOC, and reveals their mutual inhibitory effects. By separately tracking asymptomatic and symptomatic infections, model simulations identify a significant role of vaccine breakthrough in the spread of Delta. Furthermore, the severity of a Delta outbreak depends not only on the NPI and vaccination rate but also on the vaccine types. Alarmingly, despite Ontario’s existing NPI and relatively successful vaccine rollout, a future, more dangerous VOC could potentially infect a significant fraction of the province’s population and overwhelm the health care system. To stop that VOC, the province may need the simultaneous and rapid deployment of a third booster vaccine and stringent NPI.

## Introduction

In recent months, severe acute respiratory syndrome coronavirus 2 (SARS-CoV-2) variants associated with increased transmissibility have emerged and spread, one after another. The World Health Organization has designated a list of variants of concern (VOC), including Alpha (B.1.1.7), Beta (B.1.351), Gamma (P.1), Delta (B.1.617.2), and Omicron (B.1.1.529), as well as multiple variants of interest^1^. Viral lineages carrying the N501Y and/or E484K mutations, i.e., the Alpha, Beta, and Gamma variants, were first identified in Ontario, Canada in December 2020^2^. These VOC quickly outcompeted earlier SARS-CoV-2 lineages and, as of late April 2021, accounted for all new infections in Ontario, with Alpha being the most prevalent lineage^1^. Around that time, the Delta variant was first reported in the province. By 3 June 2021, the Alpha variant has been reported from at least 160 countries, Beta from 113 countries, Gamma from 64 countries, and Delta from 62 countries. Since then, the Delta variant has rapidly become the dominant circulating lineage. As of July 2021, Delta represented the majority of infections in Ontario^3^. At the time of this study, Omicron has begun spreading.

Initially, no pharmaceutical agents are known to be safe and effective at preventing or treating coronavirus disease 2019 (COVID-19)^4^. This leaves public health authorities with nonpharmaceutical interventions (NPI) as the only recourse to reduce disease transmission; these measures include bans on public gatherings, compulsory stay-at-home policies, mandating closures of schools and nonessential businesses, face mask ordinances, and quarantine. Even though the WHO initially expected vaccine development to take 18 months^5^, dedicated global efforts resulted in the approval of vaccines first in China^6^ and Russia^7^ in the summer of 2020. Near the end of that year, major vaccines developed by Pfizer-BioNTech, Moderna, and Oxford-AstraZeneca were approved in North America. Since then, the deployment of vaccines has become the most important intervention for mitigating disease severity and spread.

Despite costly NPI and the availability of vaccines, SARS-CoV-2 continues to spread and wreak havoc on a global scale. A major challenge is the rapid emergence of VOC with higher transmissivity, as noted above, and vaccine breakthrough. While major vaccines are similarly effective against the SARS-CoV-2 wild type, their efficacy against VOC is significantly less and differs among vaccine types^8^. Large discrepancy exists in vaccine deployment among countries, not only in the fraction of the population vaccinated, but also in the types of vaccines distributed. Thus, a pressing question is: *How ready are different regions of the world in stopping the spread of SARS-CoV-2 VOC?* This question must be addressed not only for existing VOC, but also for future, potentially deadlier ones. Another challenge lies in the role of asymptomatic patients, including those who are vaccinated, in spreading the disease. Asymptomatic patients may account for more than half of the COVID-19 cases^9^, and non-severe COVID-19 patients can transmit the disease regardless of their symptomatic status^10^. Given the increasing prevalence of VOC vaccine breakthrough, many of these asymptomatic patients may have been vaccinated and underestimate the likelihood of themselves being COVID-19 carriers. Thus, another critical question is: *What role do individuals with asymptomatic infections play in disease transmission?*

**Figure 1:**
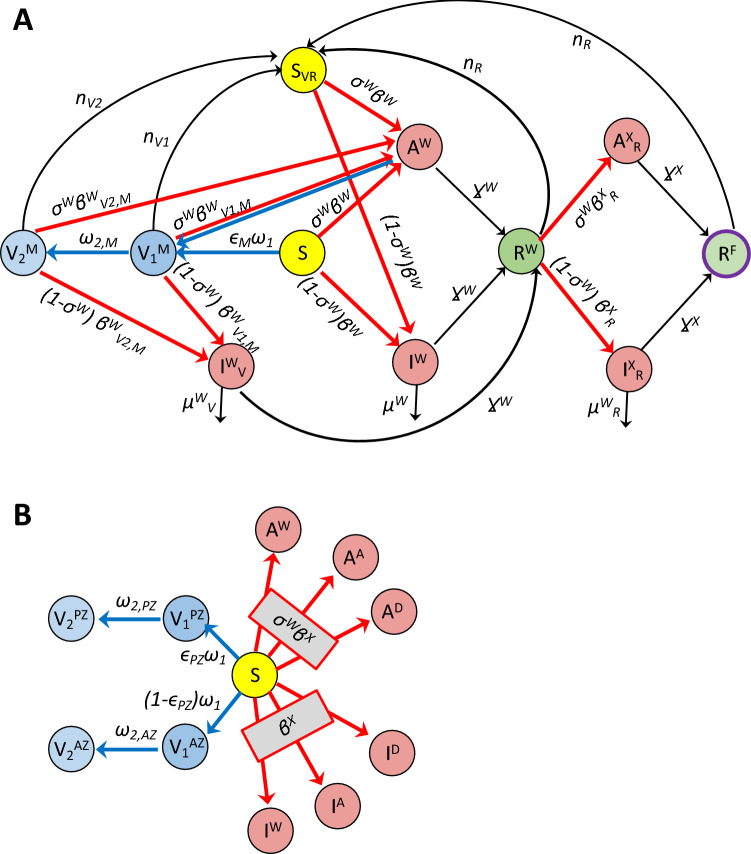
Panel (**A**), schematic diagram for a simplified version of the SV^2^(AIR)^3^ model, representing only one virus and one vaccine type. S and S_VR_, susceptible class; I^W^, I^W^_V_ and I^W^_R_, symptomatic infected class; A^W^ and A^W^_R_, asymptomatic infected class; R^W^ and R^F^ recovered class. Arrows denote the movement from one class to another, via infection (red arrows), vaccination (blue arrows), recovery, or loss of immunity. Rates are indicated on the arrows. *β,* infection rate; *Ɣ,* recovery rate; *n*, rate of immunity loss; *μ*, death rate; *ω*, vaccination rate. For a complete definition of the variables, superscripts, and subscripts, see text. In the complete model, three viruses (W = wild type, A = Alpha, D = Delta) and two vaccine types (PZ = Pfizer and AZ = AstraZeneca) are represented. Deaths are indicated only for the symptomatic infected classes, even though natural deaths are represented for all populations. Panel (**B**) shows the full connections associated with the susceptible class S. Arrows indicate movement of S into one of the six infected classes (the A’s and I’s) or one of the two vaccinated classes (V_1_’s). Movements from one of these infected or vaccinated into another class are not shown.

To answer the above equations, we developed and applied a computational model of the spread of SARS-CoV-2 variants within a community. The model represents asymptomatic and symptomatic infections, and two-dose vaccinations with variable dosing intervals. Additionally, the effects of NPI are represented by scaling the disease infectivity. A schematic diagram of the model is shown in Fig. 1. A large number of epidemiological modeling studies have been published^11^, many of which are based on the Susceptible-Infected-Recovered (SIR) formulation (e.g., ^12–15^). A key feature that distinguishes the present study from existing SIR-type models in that it is the first model to represent multiple SARS-CoV-2 VOC and their competition. We refer to this model as a SV^2^(AIR)^3^ model, for its representation of two vaccine types (V^2^), asymptomatic infections (A), and three VOC (the power ‘3’ refers to the three instantiations of each infected and recovered class). The SV^2^(AIR)^3^ model can answer the question: *To what extent do the interactions and competition among VOC impact the spread of COVID-19?* An example of VOC competition at a population level is this: an individual having been infected and subsequently recovered from one strain may be partially but incompletely protected against other strains, and if infected again, they may be more likely to develop an asymptomatic infection. Given the key role of asymptomatic cases in disease transmission, a thorough understanding of VOC competition is important in determining which (costly) NPI are necessary to prevent and limit the spread of COVID-19. We apply the model to analyze VOC dynamics in the province of Ontario in Canada. Ontario has managed a reasonable deployment of vaccines, but is nonetheless on the cusp of another wave of infections due to a highly transmissible VOC such as Delta or Omicron. That said, at the time of this study, the number of Delta infections in Ontario remains substantially lower than much of the rest of the world, which makes Ontario an interesting case study.

## Results

### The dynamics of COVID-19 VOC in Ontario, Canada

Model solution was computed to simulate the spread of SARS-CoV-2 in Ontario for two years, from January 1 2020 to December 31 2021. The SV^2^(AIR)^3^ model represents the dynamics of and interactions among three SARS-CoV-2 strains: Wild type, Alpha, and Delta, denoted by superscripts ‘W’, ‘A’, and ‘D’, respectively. On January 1 2020, we assume that the entire Ontario population was susceptible, except for 15 infected symptomatic individuals (wild-type). The Alpha strain was introduced into Ontario via the entrance of 50 asymptomatic infected individuals per day for 1.5 months at the end of 2020; Delta was introduced similarly in February and March 2021 (25 asymptomatic infected individuals per day). These parameters were chosen to product simulation results that match the initial rise of each of the VOC, Vaccination became available on December 14 2020; details for vaccination rates can be found in the supplement. The Omicron strain is not represented in the model, because insufficient data is available for this strain at the time of this study. Nonetheless, a hypothetical, more transmissible VOC is simulated.

**Figure 2:**
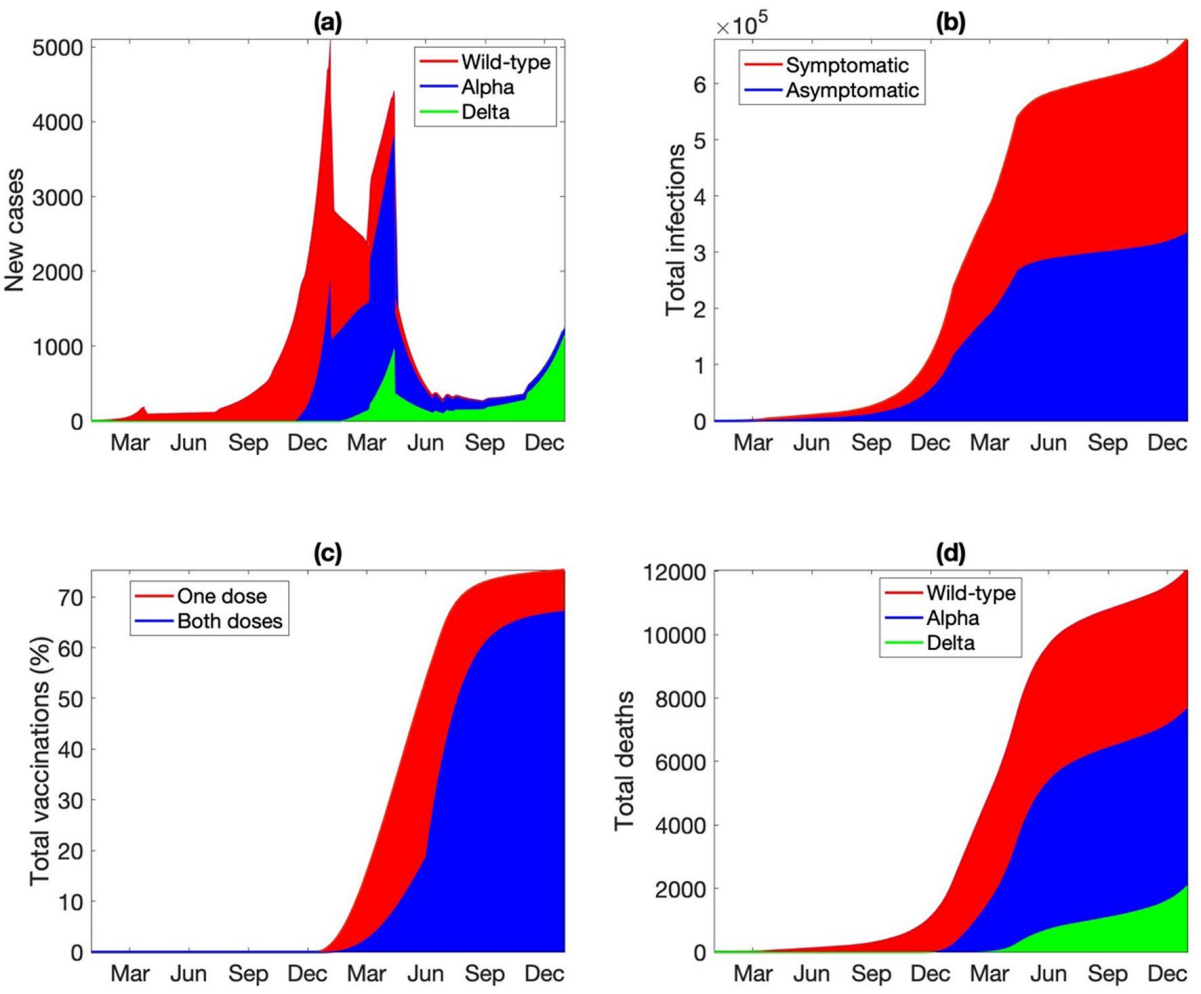
(**a**) Number of new infections for each of the viruses, shown as 7-day means from January 1 2020 to December 31 2021. (**b**) Cumulative asymptomatic and symptomatic infections for all viruses. (**c**) Fraction of population vaccinated with at least one dose and with both doses. (**d**) Cumulative deaths from each of the viruses.

Key measures of the pandemic are illustrated in Fig. 2. The total number of new infections exhibits two peaks (Fig. 2a), with the first wave in December 2020 driven by the wild type, and the second around March 2021 driven by Alpha. By mid-June 2021, the new infections are dominated by the Delta variant. These predictions are consistent with Ontario statistics (Ontario COVID-19 Science Advisory Table). The consistency between model predictions and Ontario statistics before July 2021 can be attributed to the choice of model parameters, which were based on statistics available then. Model predictions for the second half of 2021 can be used to validation. The total number of infections, including asymptomatic and symptomatic infections, are shown in Fig. 2b. The model predicts that by the end of 2021, about 680 K people would have been infected, half of whom are asymptomatic. The cumulative infection curves exhibit the steepest rise in December 2020 and March 2021, corresponding to the two peaks in the new case counts. By the end of 2021, 71% of the Ontario population would have been vaccinated with at least one dose, with 57% fully vaccinated (Fig. 2c). At that time, 11 K would have died from COVID, with 36, 47, and 17% from wild type, Alpha, and Delta, respectively (Fig. 2d). The year-end infection and death statistics are within 10% of reported data in Ontario (750 K infections and 10.2 K deaths)^16^.

**Figure 3:**
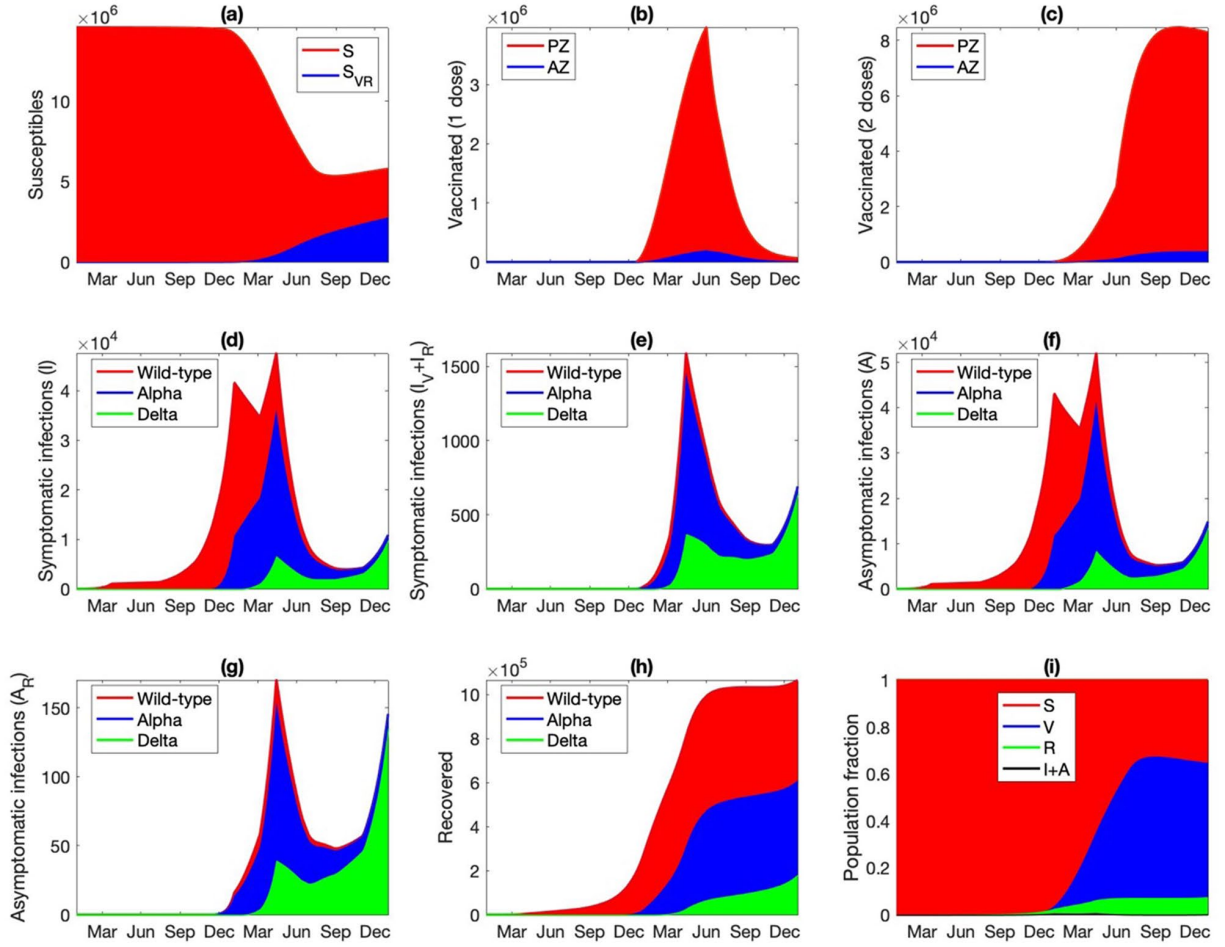
Simulated population dynamics from January 1 2020 to December 31 2021. (**a**) Susceptible individuals, original population and those who lost their immunity acquired either from an infection or vaccination. (**b**) Vaccinated with only one dose, with either Pfizer (PZ) or AstraZeneca (AZ). (**c**) Fully vaccinated. (**d**) Symptomatic infections of susceptible individuals. (**e**) Symptomatic infections of vaccinated or recovered individuals. Asymptomatic infections of (**g**) susceptible or vaccinated individuals and (**h**) recovered individuals. (**i**) Predicted fraction of population being susceptible (original or after losing immunity), vaccinated (one or two doses), recovered, and infections (symptomatic or asymptomatic).

Predicted population dynamics are shown in Fig. 3. The drop in the susceptible population becomes significant after December 2020 (Fig. 3a) and is driven primarily by the vaccine deployment. By the end of June 2021, only 37% of the Ontario population are susceptible, with the majority having received at least one dose of vaccine (Fig. 3b,c). The number of active infections peaks at the end of 2020, followed by an even higher peak at the end of spring (Fig. 3d). These are the wild-type and Alpha-driven waves identified in Fig. 2a and described above. The Alpha-driven infection peak accounts for 0.7% of the total population, but subsides as the vaccination effort accelerates. The infections are split approximately half-half between asymptomatic and symptomatic ones, with the majority unvaccinated (Fig. 3d–g). As the infection spreads, the recovered population also increases, to 7% of the population at the end of 2021 (Fig. 3h). These dynamics are summarized as fractions of the population that are susceptible, vaccinated, infected, and recovered, as shown in Fig. 3i.

### Sensitivity analysis

To identify the strongest determinants of the pandemic severity, we assess the sensitivity of model predictions to variations in key model parameters. We first conduct a sensitivity analysis in a simplified model that represents only the wild-type SARS-CoV-2, for the year 2020, but with infectivity β^W^ elevated to 5E−9. The Alpha and Delta strains are not included. We assume that NPI begin on March 1 2020, with the restrictiveness index λ taken to be uniformly 0.5 (i.e., infectivity β’s halved) for simplicity. Vaccination begins on April 1 2020 (not the end of 2020, for this simplified one-year simulation), with baseline dosing interval taken to be 4 weeks for Pfizer and 8 weeks for AstraZeneca (dosing interval is one of the parameters varied)^17^. Parameters that are varied include those that describe the clinical features of the viruses as well as provincial policy and pharmaceutical intervention.

**Figure 4:**
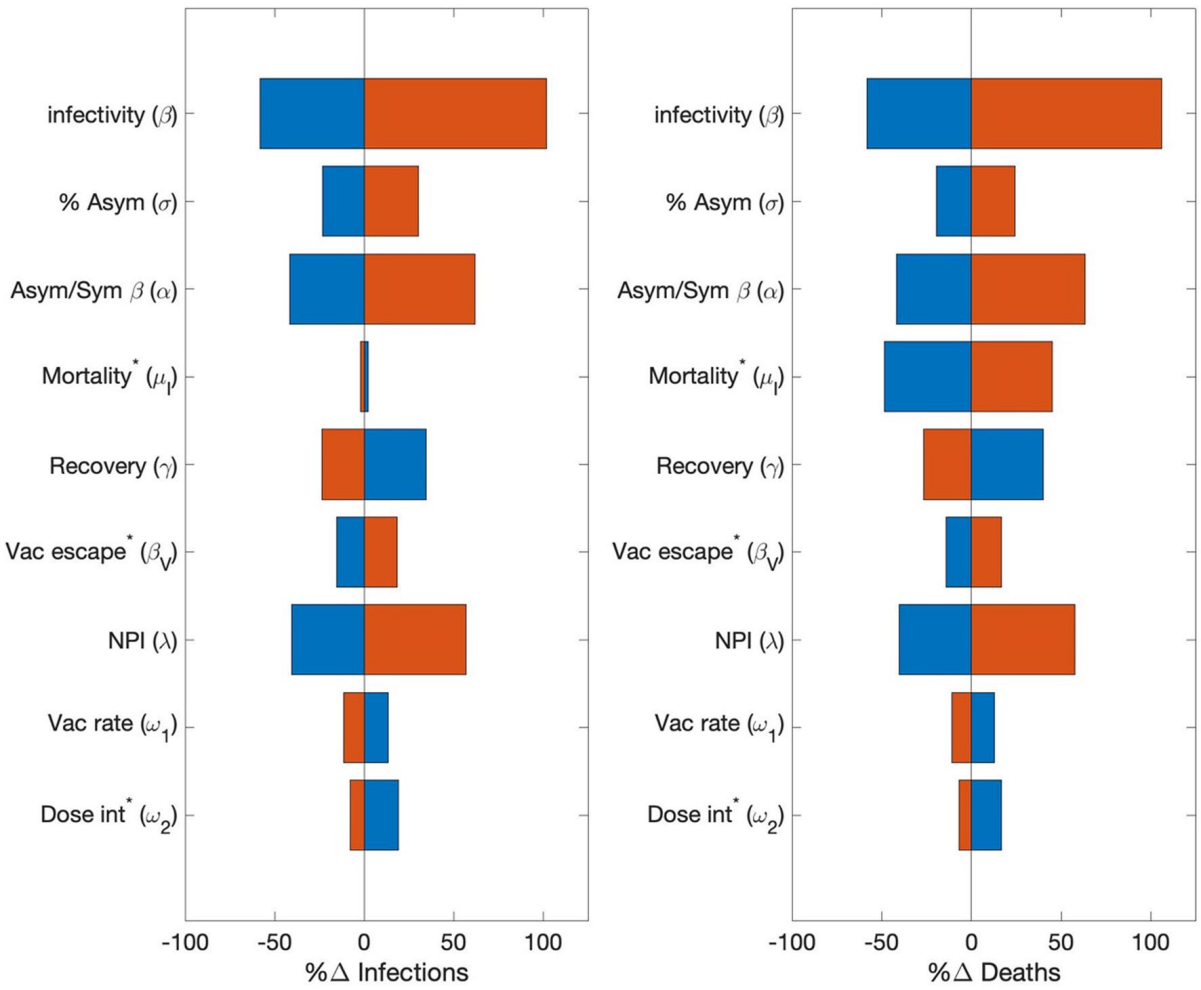
Sensitivity of model predictions, measured by total infections and deaths, to variations in model parameter values, in a one-year simulation (2020) of wild-type viral dynamics. Parameters examined include virus infectivity (β), ratio of asymptomatic versus symptomatic infections (σ), degree of increased infectivity β (due to mobility) of asymptomatic infected individuals compared to symptomatic ones, mortality rate of infected individuals (μ_I_), disease recovery rate (γ), vaccine escape rate (β_V_), NPI stringency (λ), dosing interval (ω_2_), vaccination rate (ω_1_). Asterisk (*) denote parameters that were varied by 50%; other parameters were varied by 5%. Red bar, parameter value increased; blue bar, decreased.

Clinical features of the viruses that were considered include its infectivity (β^W^), the fraction of infections that are asymptomatic (σ^W^), the infectivity ratio between asymptomatic and symptomatic individuals (α^W^), mortality rate (μ^W^), and recovery rates (γ^WA^ and γ^WI^, varied simultaneously). Recall the baseline assumption that asymptomatic individuals are assumed to be more active and 3 times more likely to spread the disease than symptomatic ones (α^W^ = 3). Thus, the number of infections directly depends on, and is in fact highly sensitive to, β^W^, σ^W^, α^W^, and also to the recovery rate γ^W^, which impacts how many new infections an infected individual can cause. Small variations in these four parameters (5%) result in changes in the number of infections and deaths that are an order of magnitude larger; see Fig. 4. COVID-19 mortality rate μ^W^_I_ affects the number of infected individuals who are alive and can participate in the spread of the disease. Because COVID-19 mortality rate can vary substantially depending on health care infrastructure, we varied μ^W^_I_ by 50%. As expected, changes in μ^W^_I_ are immediately reflected in the number of deaths. However, compared to other clinical features of SARS-CoV-2, the mortality rate has a relatively small effect on the infected population, since < 5% of them die (Fig. 4).

Provincial policy and pharmaceutical interventions that were considered include NPI that reduce the effective disease infectivity (via scaling by λ), vaccination rate (ω_1_), delay between the first and second vaccine doses (ω_2,PZ_ and ω_2,AZ_, varied simultaneously), and vaccine escape rate (β^W^_V_). λ and ω_1_ are varied by 5%. Since NPI are modelled by scaling β^W^ directly, they have large effects on model predictions (Fig. 4). Vaccination rate has lesser but still significant effects on the spread of the disease. The effects of dosing interval (ω_2_) and vaccine escape rate (β^W^_V_) are much smaller. Because dosing interval can vary greatly within the population, and because vaccine escape rate is not sufficiently well characterized, these two parameters were varied by 50%. The vaccine-related results suggest that among these three factors, an effective vaccine deployment (captured in ω_1_) is the key in combating a pandemic, and success may be achieved even by means of a less-than-ideal vaccine with higher escape rate (Fig. 4). Since the predicted numbers of infections and deaths are substantially more sensitive to variations in ω_1_ than ω_2_, if vaccine supply is limited, prioritizing the first dose by lengthening the dosing interval would be advisable, as was done in Canada.

**Figure 5:**
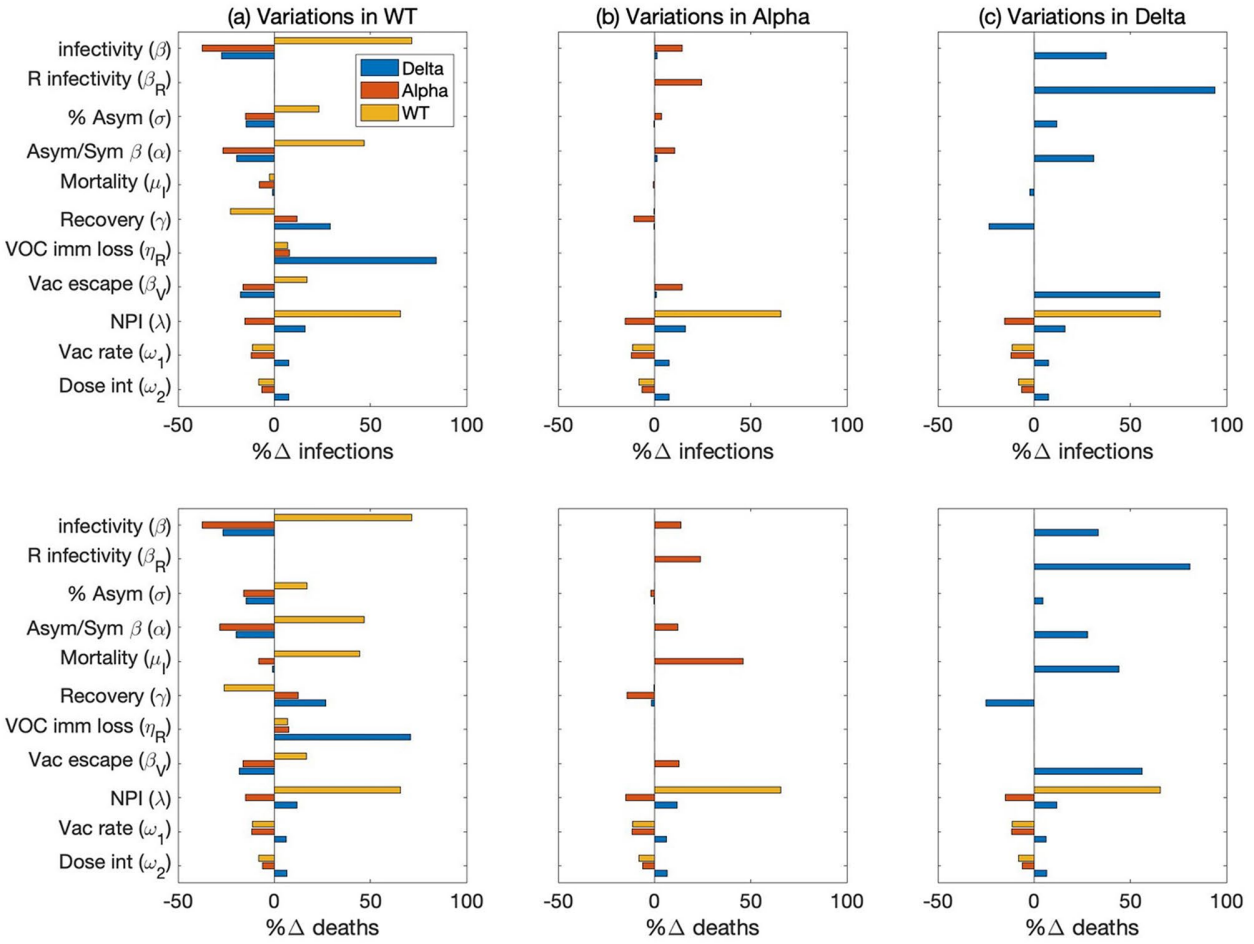
Sensitivity of model predictions, measured by total infections and deaths, to increases in model parameter values, in a two-year simulation interacting VOC dynamics. Parameters are varied individually for wild-type (column a), Alpha (column b), and Delta (column c). Relative changes in the number of infections and deaths are then computed for wild-type (yellow bars), Alpha (red bars), and Delta (blue bars). Parameter examined include virus infectivity (β), rate of infection of individuals recovered from a different variant (β_R_), ratio of asymptomatic versus symptomatic infections (σ), degree of increased infectivity β (due to mobility) of asymptomatic infected individuals compared to symptomatic ones, mortality rate of infected individuals (μ_I_), disease recovery rate (γ), rate of loss of immunity due to infection (η_R_), vaccine escape rate (β_V_), NPI stringency (λ), dosing interval (ω_2_), vaccination rate (ω_1_).

In a second sensitivity analysis, we considered the full model that represents all three variants, using the baseline parameter values (Table S1). Key model parameters were increased to determine the effects on the number of infections and deaths associated with each variant. In addition to parameters considered in the above analysis, we also considered two parameters that mediate VOC interactions: β^X^_R_, rate of infection of individuals with partial immunity from having recovered from a different variant, and η_R_, rate of loss of immunity due to infection. Some of these parameters have distinct values for each of the variants. Simulations were conducted by varying each of the parameter values for individual variants separately. Specifically, β^X^ (X = W, A, or D), σ^X^, α^X^, γ^X^, and ω_1_ were increased by 5%, λ by 10%, and μ_I_, β^X^_V_, and ω_2_ by 50%. β^X^_R_ was increased 4 folds to assess the scenario in which recovery from one variant offers only limited protected from other VOCs. η^X^_R_ was doubled to test the effect of a shorter immunity period. Sensitivity results are summarized in Fig. 5.

The effects of varying parameters associated with a given variant on the spread of that variant are similar to the single-variant analysis; compare Figs. 4 and 5. Thus, we will discuss cross-variant results. As can be seen in Fig. 5, most wild-type specific parameters have opposite effects on wild-type and on the other variants (β^X^, σ^X^, α^X^, γ^X^, β^X^_V_). This suggests that promoting the spread of the wild-type variant protects the population from Alpha and Delta, due to the partial natural immunity individuals acquire after recovering from wild-type infections. In contrast, the spread of Alpha or Delta has no effect on wild-type. That is because by the time either Alpha or Delta became rampant, the number of new wild-type cases has become negligible. β^W^_R_ (natural immunity against the wild-type strain, acquired from a Alpha or Delta infection) has essentially no effect because during the spread of the wild-type virus, no one has recovered from a different variant. In contrast, lowering the natural immunity acquired from recovery from a different variant (i.e., increasing β^A^_R_ or β^D^_R_) significantly increases the spread of Alpha or Delta. When an individually loses their naturally acquired immunity, they are susceptible to all variants. Thus, increasing the rate of loss of naturally acquired immunity (η^X^_R_) increases the spread of all three variants.

Note that λ, ω_1_, and ω_2_ are not variant specific. Their effects on the spread of the wild-type virus have been discussed above in the single-variant analysis. Due to VOC interactions, the effects of varying these parameters on the spread of Alpha and Delta are less straightforward. Reducing the severity of the NPI (i.e., increasing λ) has competing effects on the spread of Alpha and Delta: one directly from the variation in NPI, the other from the enhanced spread of the wild-type virus. These competing effects result in opposite effects on Alpha and Delta; see Fig. 5. The opposite effects of increasing ω_1_, and ω_2_ on the spread of Alpha and Delta can be explained similarly. Competition among the VOC is further considered below.

### Competition among VOC

**Figure 6:**
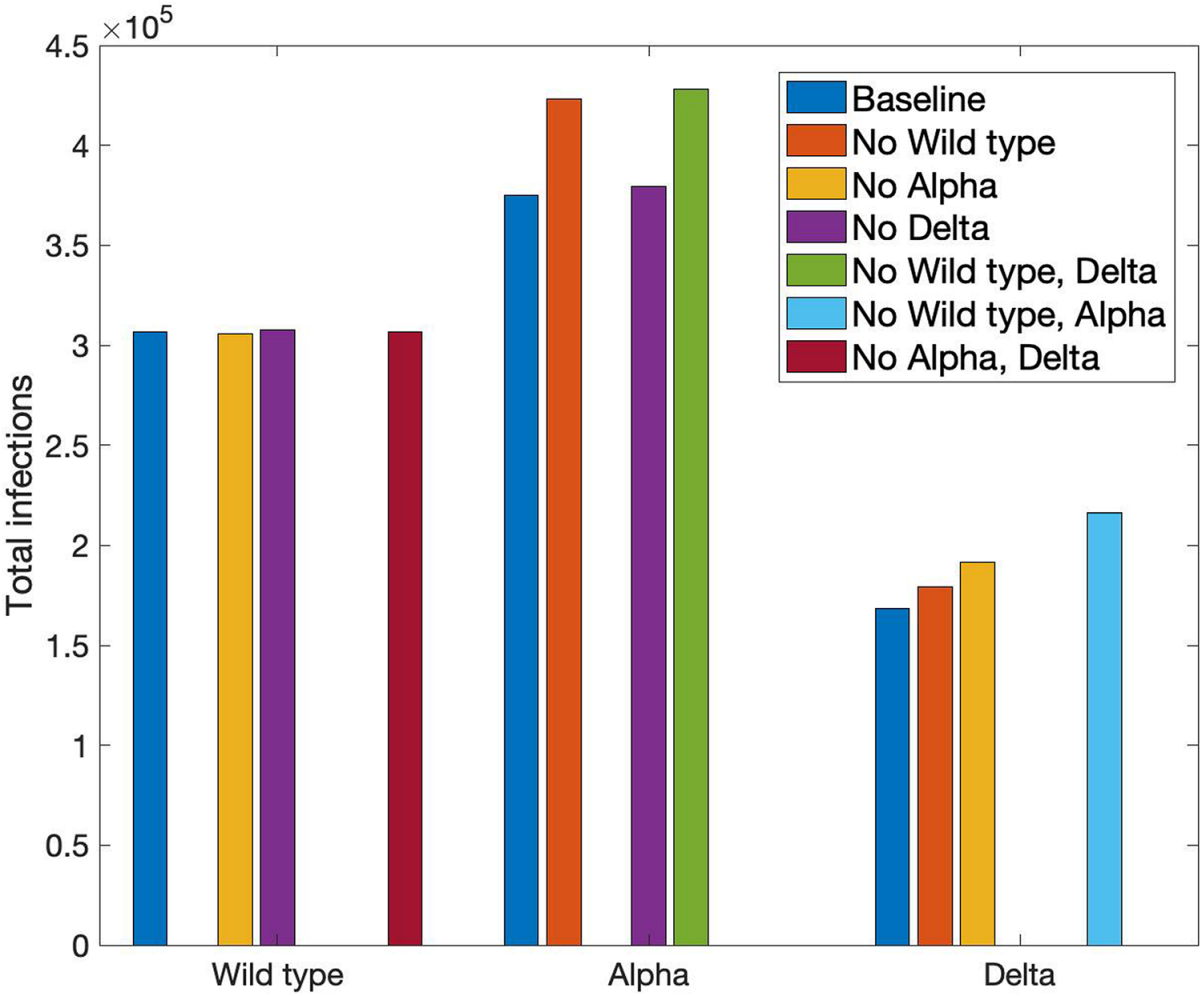
Predicted number of COVID-19 infections when some of the other variants are not in play. Results suggest that wild type limits the spread of Alpha; wild type and Alpha together limit Delta.

Individuals who have recovered from COVID-19 gain partial immunity not only to that particular strain, but other strains as well, albeit to a lesser extent. In that sense, the VOC don’t spread independently but may instead exhibit inhibitory effect on each other. To understand their interactions, we conduct simulations with some of the VOC eliminated. The predicted number of infections for each scenario is shown in Fig. 6. Results suggest that Alpha and Delta have minimal impact on the spread of the wild type. When Alpha and/or Delta were eliminated, the number of wild-type infections was barely affected. That insensitivity can be explained by the observation that the wild-type strain spread with little competition for almost a year. By the time the other VOC emerge, the wild type is already on its decline due to the NPI. In contrast, the wild type has a significant impact on both Alpha and Delta. In the absence of the wild type, fewer of the population would have acquired partial immunity to Alpha, and the number of Alpha infections would be 19% higher. Similarly, in the absence of both the wild type and Alpha, there would have been 35% more Delta infections. Similar trends are seen in the mortality counts (results not shown). These results point to the importance of taking VOC competition into account when analyzing and predicting COVID-19 dynamics.

### Spread of the Delta variant

The Delta variant, which compared to earlier strains exhibits substantially higher transmissivity and vaccines breakthrough rate, drove a surge in COVID-19 cases in much of the world. The baseline model simulation describes what happened in Ontario: that after its introduction at the beginning of 2021, the number of new Delta cases peaked in April, before it was suppressed by the provincial lockdown that began on April 13. Simulation predicts that provided that some significant NPI remain in place throughout the remainder of 2021 and 2022 (λ ≤ 0.4, except during the winter holidays), the Delta variant may remain under control. However, given the high price that society must pay for being insufficiently vigilant against COVID-19, it is prudent to explore all possibilities in which the pandemic can explode. Hence, we conduct simulations to analyze the spread of the Delta variant under alternative scenarios.

**Figure 7:**
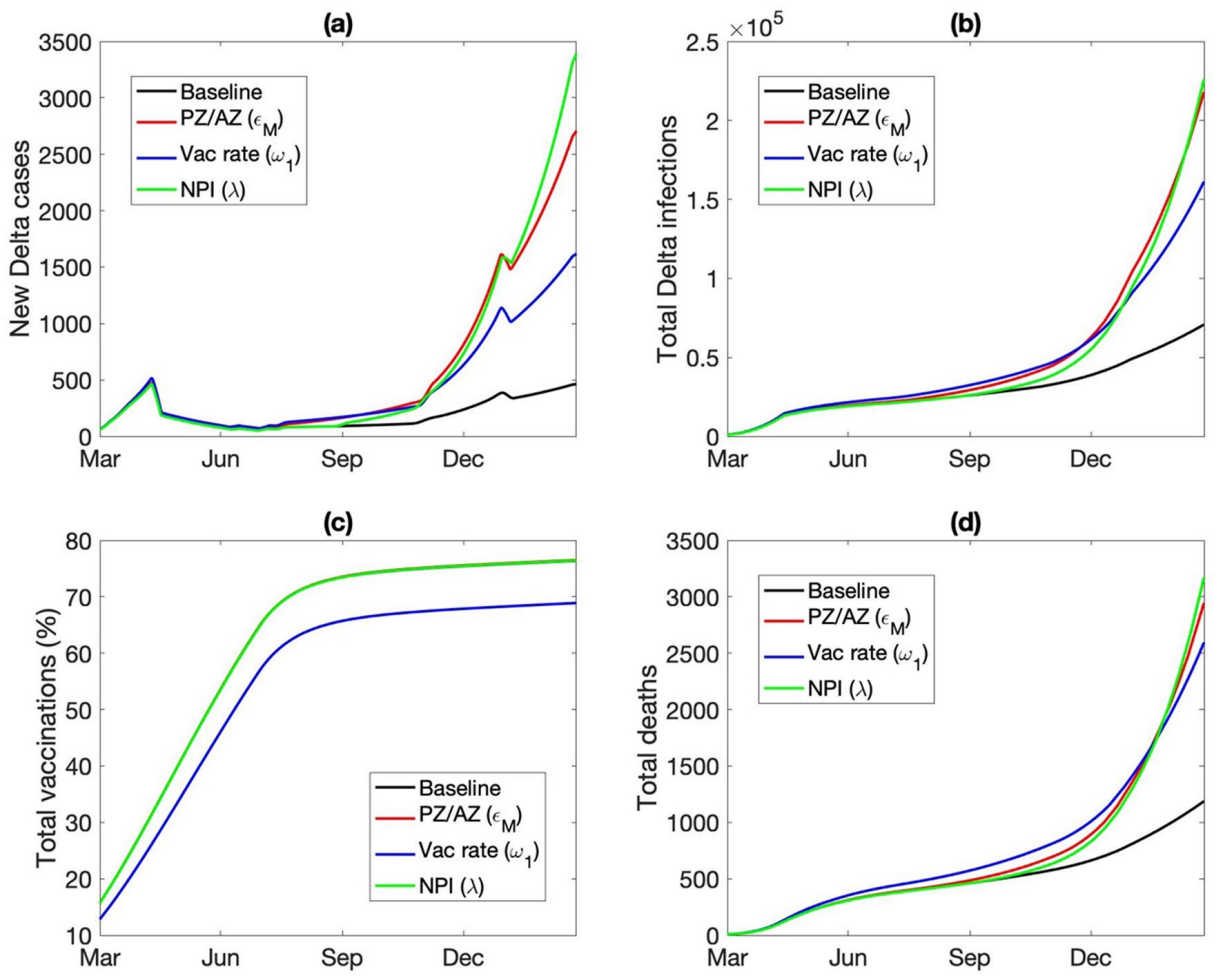
Predicted spread of the Delta variant, from March 2021 to March 2022, obtained for baseline parameters and three alternative scenarios. “PZ/AZ,” lower fraction of vaccines being Pfizer (from 95 to 40%), with the reduction made up with AstraZeneca, which offers reduced protection from Delta infections and a longer dosing delay. “Vac rate,” vaccination rate reduced by 20%. “NPI”, less stringent lockdown measured, with mobility increased by 25%. Among these scenarios considered, Delta outbreak is the most severe when the majority of the vaccines are AstraZeneca.

The baseline model assumes that 95% of the vaccines are Pfizer and 5% are AstraZeneca, consistent with Ontario vaccination statistics. What if a larger fraction of the vaccines were AstraZeneca, as is the case in the UK^18^. A notable difference between the two vaccine types is that the Pfizer vaccine offers stronger protection than AstraZeneca against all COVID variants, especially Delta, after either one or two doses. For instance, an individual having received both doses of the Pfizer vaccine is 88% protected against the Delta variant, whereas with the AstraZeneca vaccine the protection is only at 60%^19^. We conduct a simulation in which only 40% of the vaccines are Pfizer. With a larger portion of the vaccinated population now under reduced vaccine protection, the spread of the VOC is substantially accelerated. By the end of 2021, the model predicts 794 new Delta infections a day, more than three times higher than the baseline prediction of 234 new Delta infections a day (Fig. 7a). The total number of Delta infections and related deaths are also significantly higher, predicted to be 62 K and 882 at the end of 2021, respectively, compared to baseline values of 39 K and 689 (Fig. 7b,d).

If the provincial vaccination rate were lower, such that by September 1 2021 only 67% of the population are vaccinated with at least one dose, compared to the baseline vaccinated percentage of 74% (Fig. 7c), the total number of Delta infections and deaths would increase by 75% by the end of March 2022; see Fig. 7b,d.

Beginning mid-June 2021, Ontario began to gradually reopen, with in-person classes set to resume in September. The baseline model assumes that the NPI that remain will still reduce COVID-19 infectivity by 60%. What if that assumption is overly optimistic? Indeed, it is not uncommon to experience pandemic fatigue and crave personal interactions; also, there may be outbreaks in schools. Thus, we consider a scenario with less stringent NPI starting from the fall of 2021 (September 1 2021), with λ increased from its baseline value of 0.4 to 0.5 onwards. The model predicts the reduced NPI would accelerate the spread of Delta, such that by March 2022, the total number of Delta infections would be 50% higher than baseline (Fig. 7b).

**Figure 8:**
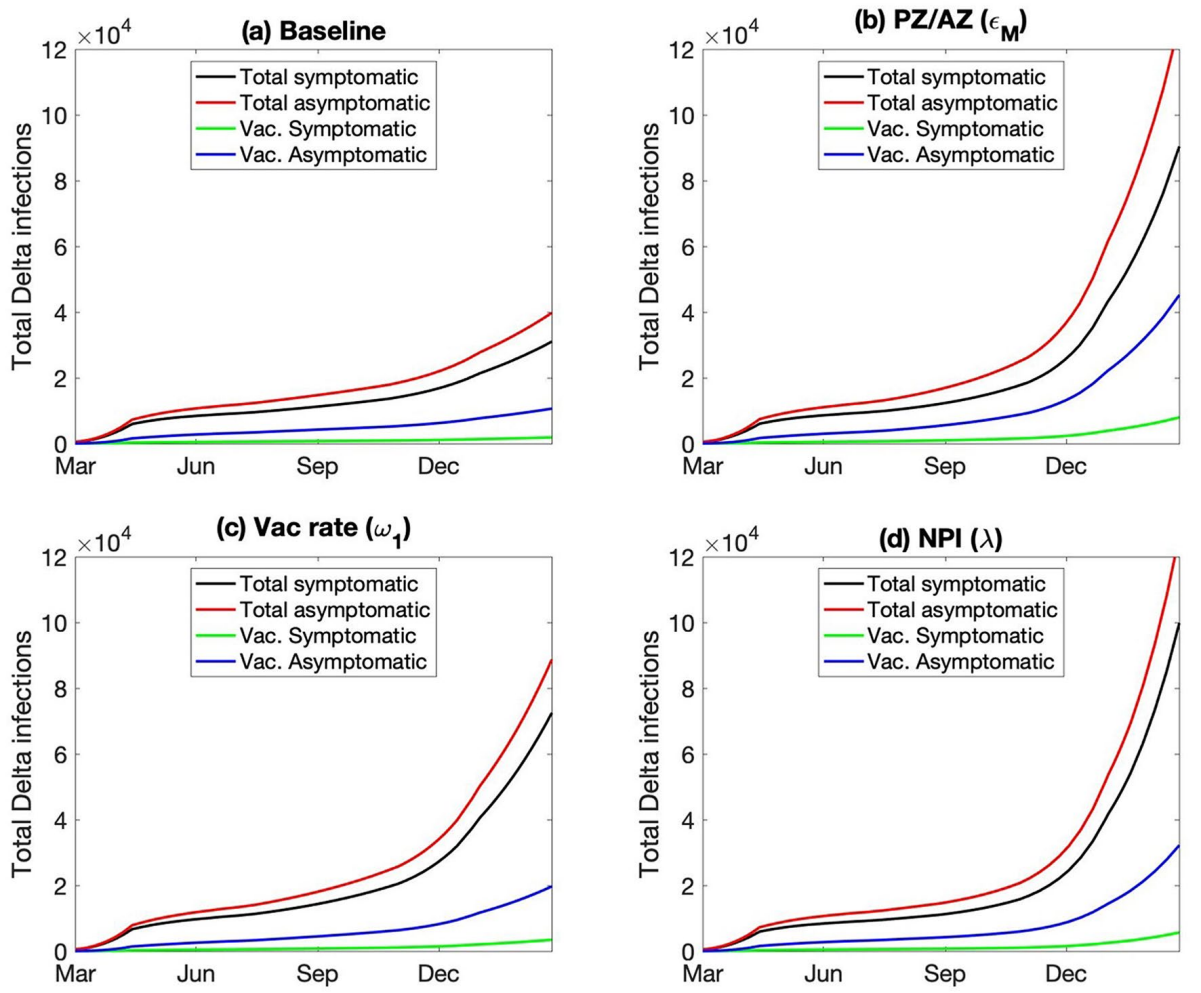
Number of different types of Delta infections: total symptomatic and asymptomatic infections, and those of vaccinated individuals. In all four scenarios, asymptomatic patients and vaccinated patients account for a significant fraction of the disease carriers.

Who are getting infected by the Delta variant, and who are the carriers? Interestingly, while the wild-type infections are about equally split between asymptomatic and symptomatic, ~ 60% of the Delta-infected population are asymptomatic. This difference may be attributable to the vaccination status of the infected population. Due to the larger vaccine breakthrough rate of the Delta variant, almost a quarter of the infected individuals have been vaccinated (Fig. 8a); in contrast, for the wild type, vaccinated individuals make up a negligible fraction of the infected population (Fig. 3). Because vaccination protects one from most major COVID-19 symptoms, most of the vaccinated infections are assumed to be asymptomatic (85% compared to 50% for the unvaccinated). Taken together, the asymptomatic patients, including those who are vaccinated, play a more significant role in spreading the Delta variant, compared to the wild type. And that difference is even larger in the alternative scenario where most of the vaccines are AstraZeneca (Fig. 8b).

### The spread of a hypothetical, deadlier VOC, Neos

Alpha is more infectious than the wild type; Delta is worse than Alpha; Lambda may be even worse. How bad can things get? What are the effective and reasonable measures against the challenges presented by an ever-evolving coronavirus? To gain insights, we simulate a dangerous hypothetical variant, which we refer to as “Neos.” Neos is designed to be deadlier by increasing its (i) infectivity (14% higher than the Delta variant), (ii) vaccine escape rate (70% for one dose of Pfizer or AstraZeneca, similar to Delta which is 67%; 25% and 50% for two doses of Pfizer and AstraZeneca, respectively; compared to 12% and 40% for Delta), and (iii) fraction of asymptomatic infections (55% instead of 50%). Recovery rate, mortality rate, and other clinical features are assumed to be the same as the wild type.

**Figure 9:**
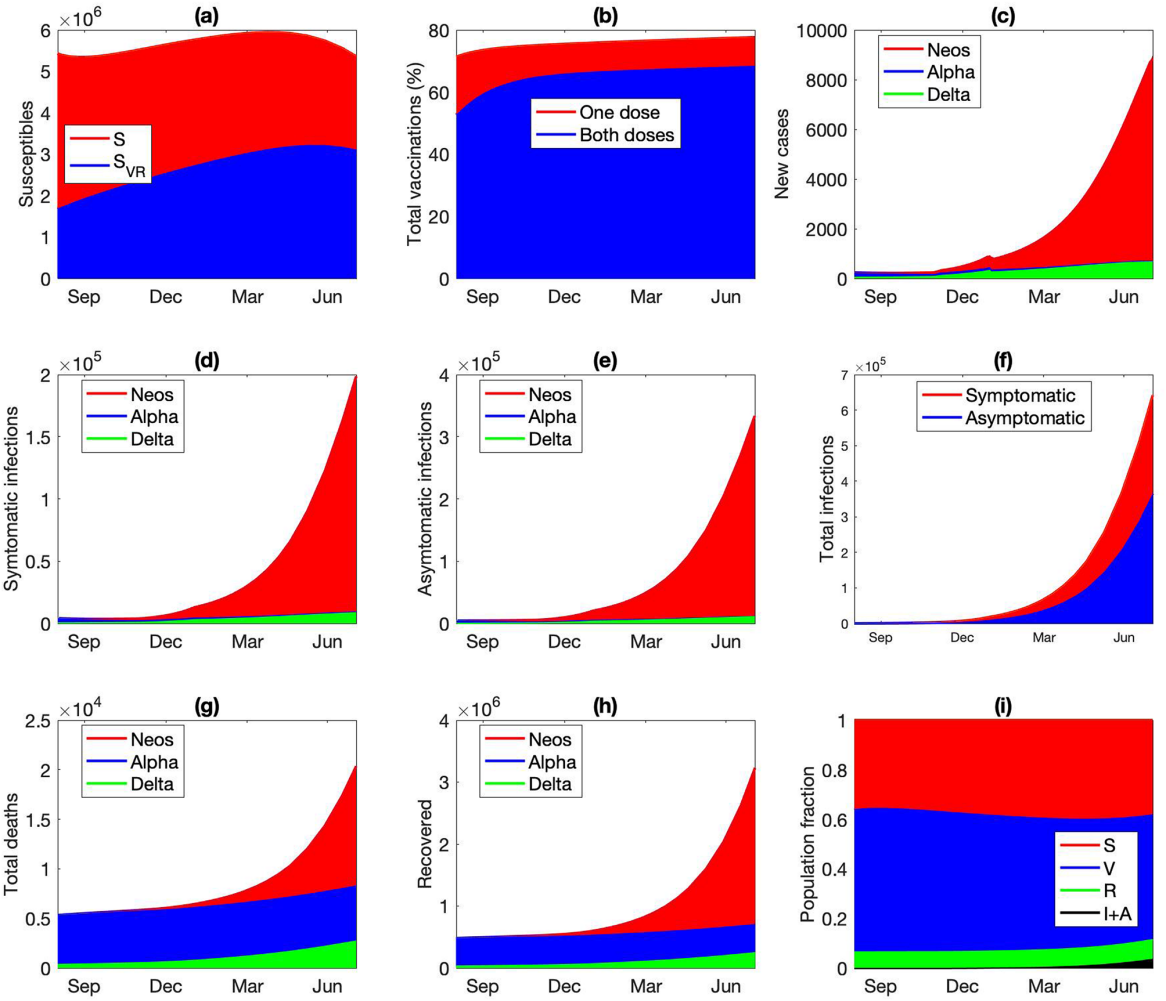
Dynamics of hypothetical VOC (“Neos”), simulated from August 1 2021 to June 31 2022. (**a**) Susceptible individuals, original population and those who lost their immunity acquired either from an infection or vaccination. (**b**) Vaccinated with only one dose or both, with either Pfizer (PZ) or AstraZeneca (AZ). (**c**) New infections per day (Neos, Alpha, Delta) since August 1 2021. (**d**) Total symptomatic infections since August 1 2021. (**e**) Total asymptomatic infections since August 1 2021. (**f**) Cumulative infections since August 1 2021. (**g**) Cumulative deaths from Neos, Alpha, and Delta. (**h**) Total number of individuals having recovered from Neos, Alpha, and Delta infections. (**i**) Fraction of population being susceptible, vaccinated (one or two doses), recovered, and infections (symptomatic or asymptomatic).

We assume that Neos emerges in the fall of 2021. The predicted population dynamics that describe its spread, from August 1 2021 to June 30 2022, is shown in Fig. 9. After its introduction, Neos quickly takes hold due to its high transmissivity, and by the end of 2021, it has overtaken Delta to account for the majority of new infections (Fig. 9c). By mid 2022, the infected individuals would account for 1% of the entire population (Fig. 9f), despite nearly 80% of the population having been vaccinated. More than 6.6 K would have died from Neos (Fig. 9g); that is likely an underestimate, since the number of severe cases might overwhelm the hospitals, elevating the mortality rate.

**Figure 10:**
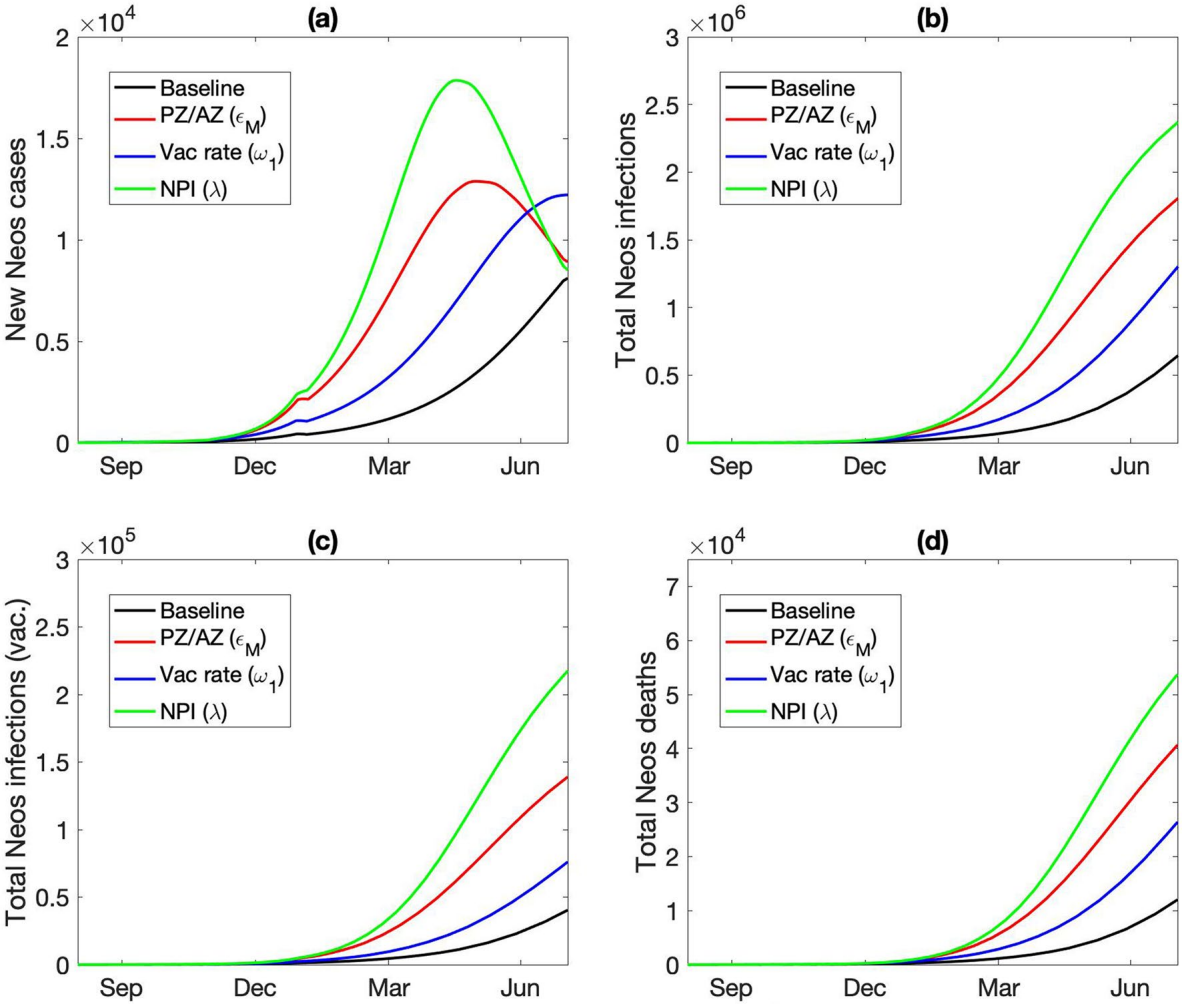
Predicted spread of hypothetical VOC “Neos,” shown from August 1 2021 to June 31 2022. Results are obtained for baseline parameters and three alternative scenarios. “PZ/AZ,” lower fraction of vaccines being Pfizer (from 95 to 40%), with the reduction made up with AstraZeneca, which offers reduced protection from Delta infections and a longer dosing delay. “Vac rate,” vaccination rate reduced by 20%. “NPI”, less stringent lockdown measured, with mobility increased by 25%. (**a**) Number of new Neos infections. (**b**) Total number of Neo infections. (**c**) Total number of vaccinated individuals infected with Neos. (**d**) Cumulative dealths from Neos infections.

While those predicted numbers are alarming, the situation could be even more dire. How much more quickly would Neos spread under the three alternative scenarios we previously considered for Delta? That is, (i) if 60% of the vaccines are AstraZeneca, (ii) if vaccination deployment is slower, and (iii) if NPI are relaxed. If the vaccination rate is 20% lower, the model predicts that the total number of Neos infections would be 3 times the original value; with 60% AstraZeneca vaccines, 4.6 times; fewer restrictions (λ = 0.5 instead of 0.4), 5 times. See Fig. 10b. Similar trends are predicted for the vaccinated individuals infected by Neos (Fig. 10c), and for the total deaths from the Neos strain (Fig. 10d). In the case where NPI are relaxed, by the middle of 2022, 9% of the entire population would have been infected by Neos, and 0.3% would have died from the disease, not counting other strains.

**Figure 11:**
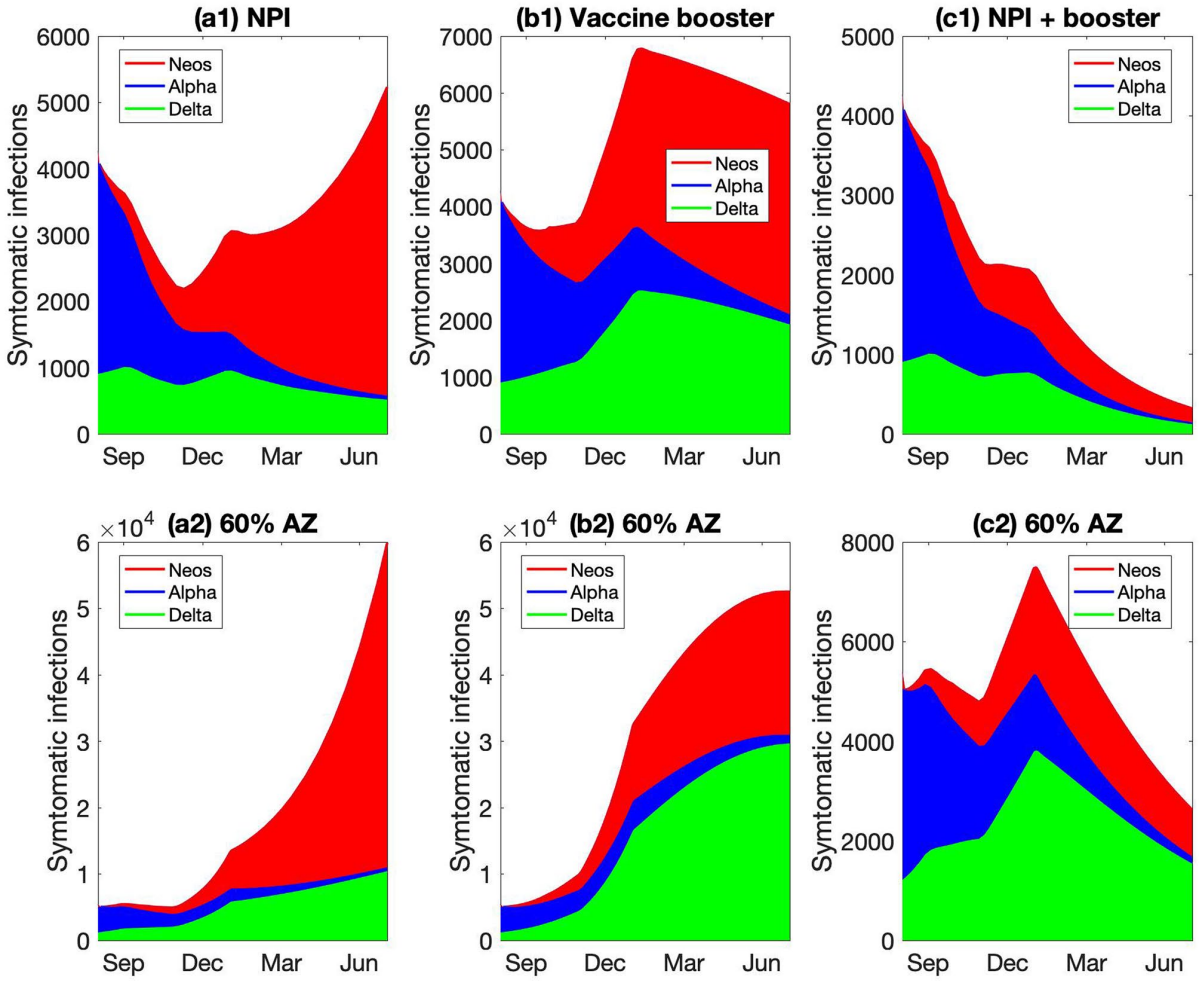
Predicted spread of hypothetical VOC “Neos,” shown from August 1 2021 to June 31 2022. Results are obtained for three scenarios: (**a1**) more stringent NPI, with mobility decreased further by 40% starting September 1 2021; (**b1**) a third vaccine booster that offers twice the protection of two doses; (**c1**) a combination of NPI and vaccine booster. Analogous simulations are then conducted with 60% of the vaccines being AstraZeneca instead of 5% (**a2**) to (**c2**). Only by combining pharmaceutical and NPI can the spread of Neos be stopped.

What can the province to do combat the spread of Neos? Two potential measures are investigated. First, we consider more stringent NPI, perhaps a provincial lockdown. As noted above, the baseline model includes some NPI that are assumed to lower social contacts by at least 60%. We evaluate the effectiveness of additional NPI that further limit social contacts, and thus infectivity of all variants, by another 60%. These measures are assumed to commence on September 1 2021 and remain in place through the end of the simulation. The predicted number of symptomatic infections from Neos, Alpha, and Delta are shown in Fig. 11a1. We choose to highlight symptomatic infections because they strongly correlate with stress on the healthcare system. During the simulated period, the wild-type has essentially disappeared and is thus not shown. The model predicts that such stringent measures may slow the spread of Neos. The number of Neos infections still increases, but at a drastically lower rate than in the original setting (compare Figs. 9d and 11a1).

Many stringent NPI are associated with high economic costs. And the model predicts that even stringent NPI may not eradicate Neos. Thus, we consider a pharmaceutical measure: a vaccine booster. A third Pfizer shot has been reported to offer increased protection from the Delta variant and potentially other VOC^20^. In this simulation, we assume that a third dose of either the Pfizer or AstraZeneca vaccine would reduce vaccine breakthrough of Neos to 10% and 20%, respectively (compared to 25% and 50%, respectively, with two doses), and also double the protection against Alpha and Delta. To model a third vaccine dose, we add a new population **V**_**3**_^**M**^ that has received three vaccine shots.

1 $$\frac{d{V}_{3}^{M}}{dt}={\omega }_{2}^{M}{V}_{2}^{M}-{V}_{3}^{M}\left(\sum_{X=W,A,D}{\beta }_{V3,M}^{X}\left({I}^{X}+{\alpha }^{X}{A}^{X}\right)\right)-\left({\eta }_{V3}+\mu \right){V}_{3}^{M}$$

The model predicts that the third vaccine dose, if indeed effective, would be successful in suppressing the spread of Neos; see Fig. 11b1. During 2022, the number of active COVID-19 cases steadily decreases, driven primarily by the decline in Alpha and Delta infections, even as Neos infection numbers remain relatively stable.

While neither NPI or a vaccine booster alone can rapidly eradicate Neos, implemented together that goal can be accomplished. A model that combines the two measures predicts that the number of symptomatic COVID-19 cases would decrease throughout the simulated period to fewer than 200 cases in mid 2022; see Fig. 11c1.

How effective are these measures in a region with 60% AstraZeneca vaccines? Model simulations predict that with NPI alone, there would still be widespread Neos infections, due to Neos’ higher vaccine breakthrough with the AstraZeneca vaccines (Fig. 11a2). With the dissemination of a third vaccine dose to increase vaccine protection, the number of Neos infections continues to increase, albeit at a slower rate, and the total symptomatic infections finally begin to decline by mid 2022 (Fig. 11b2), after exceeding 0.1% of the total population. These results suggest that neither NPI nor a vaccine booster would prevent the healthcare system from being overwhelmed. Implemented together, these two measures are sufficiently efficient to bring the total number of symptomatic COVID-19 infections to < 1000 by mid 2022 (Fig. 11c2).

## Discussion

The principal objective of the present study is to better understand the spread of the VOC of SARS-CoV-2, and how effective different interventions may be. To achieve that objective, we developed an SV^2^(AIR)^3^ model that represents factors key to the spread of COVID-19: (i) asymptomatic and symptomatic infections^21,22^, (ii) simultaneous spread of multiple VOC, (iii) two-dose vaccinations with variable dosing intervals^23^, (iv) two vaccines with different efficacy, and (v) the effects of NPI^24^. Main findings of this study include:

1. It is well understood that for any infectious disease, the major factors that determine how fast it spreads include infectivity, the extent of the NPI, and vaccination rate. For COVID-19, our sensitivity analysis reveals two additional factors, the prevalence of asymptomatic infections and the enhanced infectivity of asymptomatic patients (Fig. 4).
2. The present model is the first in capturing VOC interactions at a population level. Individuals having recovered from an infection by one strain acquire partial immunity against other strains, and if infected again, are more likely to develop an asymptomatic infection. Model simulations indicate that some VOC have an inhibitory effect on the spread of others (Figs. 5 and 6).
3. While the Delta strain is undoubtedly wreaking havoc globally, severity differs among countries. Our analysis suggests that how fast Delta spreads depends not only on the NPI and on the fraction of the population who are vaccinated, but also on the types of the vaccines distributed (Fig. 7).
4. Vaccinated individuals have a significant chance of suffering a vaccine breakthrough with Delta. Model analysis points to a significant role of the vaccinated individuals in the spread of Delta, particularly in a community where most of the vaccines are AstraZeneca (Fig. 8).
5. Given its current NPI and relatively successful vaccination rollout, is Ontario prepared for the emergence of a more dangerous VOC? A VOC that is more infectious, causes more asymptomatic infections, and has a higher vaccine breakthrough rate? Unfortunately, our model simulation suggests that the answer is no. Without additional interventions, such a VOC would infect many more and likely overwhelm the health care system (Fig. 9). The situation would likely be worse in another region that has a lower rate of vaccination or less effective vaccines (Fig. 10).
6. To stop such a dangerous VOC, one may need both simultaneous and rapid deployment of pharmaceutical and NPI (Fig. 11).

Results of this study point to the importance of sustained vigilance against SARS-CoV-2. As previously noted, a significant number of the Delta infections are caused by vaccinated individuals with asymptomatic infections. Consequently, even with > 70% of the Ontario population have received at least one vaccine dose, some NPI must remain to prevent the further spread of Delta, or the emergence and rapid spread of the next series of more dangerous VOC. When in-person classes resume in public schools and college in September, any Delta outbreak and the emergence of new VOC must be closely monitored, and if necessary, NPI must be reinstated. And for these NPI to be effective, they must begin sufficiently early.

The news that a third (or fourth) dose of the Pfizer vaccine enhances one’s protection against VOC is exciting^25^. Indeed, the combination of NPI and a booster shot may be necessary in our fight against a future, more transmissible and deadlier VOC. That said, given that much of the world have inadequate access to vaccines, the deployment of a third vaccine dose in the developed countries raises some moral questions.

### Limitations of the study

The SV^2^(AIR)^3^ model separates the population in terms of their health and vaccination status: susceptible, vaccinated (one dose or two, with Pfizer or AstraZeneca), infected (asymptomatic or symptomatic, infected by different VOC), and recovered. Each subpopulation is assumed to be homogeneous. One major limitation of the model is its lack of age structure. COVID-19 infection and mortality rates are known to exhibit distinct age and, to a lesser extent, gender specificity^26–28^, and those demographics characteristics differ among the VOC^29^. Also, age and gender determine one’s social behaviors, which would affect their susceptibility, or ability to infect others if they are sick. The impacts of NPI (e.g., school closure) would also differentially impact different age groups. The severity of COVID-19 sequela depends also on the infected individual’s health^30–32^. For more realistic simulations of COVID-19 dynamics, age, gender, and health specificity of COVID-19 infections should be incorporated into the present model by separating each subpopulation into key age groups, genders, and health conditions.

Furthermore, spatial heterogeneity likely has a significant impact on the spread of infections. As can be seen in the geographical heterogeneity in the number of COVID-19 cases in Ontario, socio-economic factors also play an important role in the local spread of COVID-19. These factors include the number of people per household, job locations, frequency of public transportation usage, regional vaccination rates, and social connectedness, etc. Different socio-economic groups are likely affected differently by the NPI. Thus, a worthwhile extension would be to divide Ontario into regions with distinct demographics, represent the spread of COVID-19 within each region using an incarnation of the SV^2^(AIR)^3^ model, and then loosely connect these sub-models based on the estimated amounts of communications mediated by job or social travels.

The present model is formulated using data from Ontario, Canada (Ontario COVID-19 Science Advisory Table). Geographical and socio-economic parameters can be refitted to simulate VOC dynamics in a different province in Canada or a different country (e.g., infectivity β, vaccine type ε, COVID-19 mortality rate μ^X^, NPI λ). These models can be a valuable decision-support tool for public health.

## Methods

### Computational model description

The SV^2^(AIR)^3^ model simulates the spread of three SARS-CoV-2 strains in Ontario: wild type, Alpha, and Delta. Population in the province is divided into several classes based on their health and vaccination status. Two “susceptible” populations are tracked: the original susceptible individuals (denoted ‘**S**’), who are unvaccinated and have never been infected, and those who become susceptible again after losing their immunity from either vaccination or infection (denoted ‘**S**_**VR**_’; more about loss of immunity below). The original susceptible population (S, but not S_VR_) vaccinate at a rate of ω_1_ and enter the **V**_**1**_^**M**^ class. Two vaccine types are represented, Pfizer-BioNTech (M = PZ) and AstraZeneca (M = AZ). Pfizer-BioNTech, which we refer to as “Pfizer,” also represents the Moderna vaccines. We denote the fraction of Pfizer vaccines by ε_PZ_ and the remainder ε_AZ_ = 1−ε_PZ_ are AstraZeneca. Those who received the first dose will receive their second dose and enter the **V**_**2**_^**M**^ class at rate of ω_2_^M^, where M denotes either PZ or AZ, as dosing interval differs between vaccine types. Mixed vaccination is not represented. Individuals vaccinated with one or two doses lose their immunity at a rate of η_V1_ and η_V2_, respectively, and enter the S_VR_ class. We assume that those who lost the immunity did so unwittingly so would not get vaccinated again.

2 $$\frac{dS}{dt}=-S\left(\sum_{X=W,A,D}{\beta }^{X}{I}_{\text{total}}^{X}+{\omega }_{1}\right)+\mu \left(N-S\right)$$

3 $$\frac{d{S}_{VR}}{dt}={\eta }_{V1}\sum_{M=PZ,AZ}{V}_{1}^{M}+{\eta }_{V2}\sum_{M=PZ,AZ}{V}_{2}^{M}+{\eta }_{R}\left({R}^{F}+\sum_{X=W,A,D}{R}^{X}\right)-{S}_{VR}\sum_{X=W,A,D}{\beta }^{X}{I}_{\text{total}}^{X}-\mu {S}_{VR}$$

4 $$\frac{d{V}_{1}^{M}}{dt}={\varepsilon }_{M}{\omega }_{1}\left(S+\sum_{X=W,A,D}{A}^{X}\right)-{V}_{1}^{M}\sum_{X=W,A,D}{\beta }_{V1,M}^{X}{I}_{\text{total}}^{X}-\left({\omega }_{2}^{M}+{\eta }_{V1}+\mu \right){V}_{1}^{M}$$

5 $$\frac{d{V}_{2}^{M}}{dt}={\omega }_{2}^{M}{V}_{1}^{M}-{V}_{2}^{M}\left(\sum_{X=W,A,D}{\beta }_{V2,M}^{X}{I}_{\text{total}}^{X}\right)-\left({\eta }_{V2}+\mu \right){V}_{2}^{M}$$

where ${I}_{\text{total}}^{X}={I}^{X}+{I}_{V}^{X}+{I}_{R}^{X}+{\alpha }^{X}\left({A}^{X}+{A}_{R}^{X}\right)$. Except for the symptomatic patients (see below), all die naturally at a rate of μ. Births are added to S at a rate of μ.

Both susceptible classes (S and S_VR_) become infected by one of the viruses at rate β^X^, potentially by coming in contact with a symptomatic infected individual (denoted **I**^**X**^), where superscript ‘X’ denotes one of the SARS-CoV-2 viruses: ‘W’ for wild-type, ‘A’ for Alpha, and ‘D’ for Delta. An infection can also be caused by an asymptomatic infected individual (denoted **A**^**X**^), with a higher rate of α^X^β^X^, where α^X^ > 1 with the assumption that asymptomatic individuals are more socially active. We assume that for a virus type X, a fraction σ^X^ of the infections are asymptomatic. Symptomatic infection is associated with a mortality rate (μ^X^) higher than natural death (μ), but asymptomatic infection does not increase one’s mortality rate. Vaccinated individuals may be infected as well, at a reduced rate of β^X^_VM_ (< β^X^), where V is either V1 or V2, and M denotes the vaccine type. Asymptomatic infections of vaccinated individuals enter **A**^**X**^ class, same as infections of unvaccinated individuals. In contrast, symptomatic infections of vaccinated individuals are assumed to have a better survival rate (with a mortality rate μ^X^_V_ < μ^X^), and are represented as a separate infected class **I**^**X**^_**V**_. Unvaccinated asymptomatic patients vaccinate at the same rate as the susceptible population (ω_1_).

6 $$\frac{d{I}^{X}}{dt}=\left({1-\sigma }^{X}\right)\left(S+{S}_{VR}\right)\left(\sum_{X=W,A,D}{\beta }^{X}{I}_{\text{total}}^{X}\right)-\left({\gamma }^{X}+{\mu }^{X}\right){I}^{X}$$

7 $$\frac{d{I}_{V}^{X}}{dt}=\left({1-\sigma }^{X}\right)\left(\sum_{M=PZ,AZ}\left({V}_{1}^{M}\sum_{X=W,A,D}{\beta }_{V1,M}^{X}{I}_{\text{total}}^{X}\right)+\sum_{M=PZ,AZ}\left({V}_{2}^{M}\sum_{X=W,A,D}{\beta }_{V2,M}^{X}{I}_{\text{total}}^{X}\right)\right)-\left({\gamma }^{X}+{\mu }_{V}^{X}\right){I}_{V}^{X}$$

8 $$\frac{d{I}_{R}^{X}}{dt}=\left({1-\sigma }^{X}\right){\beta }_{R}^{X}{I}_{\text{total}}^{X}\left(\sum_{Y\ne X}{R}^{Y}\right)-\left({\gamma }^{X}+{\mu }_{V}^{X}\right){I}_{R}^{X}$$

9 $$\frac{d{A}^{X}}{dt}={\sigma }^{X}\left(S+{S}_{VR}\right)\left(\sum_{X=W,A,D}{\beta }^{X}{I}_{\text{total}}^{X}\right)+{\sigma }_{V}^{X}\left(\sum_{X=W,A,D}\left({\beta }_{V1}^{X}{V}_{1}+{\beta }_{V2}^{X}{V}_{2}\right){I}_{\text{total}}^{X}\right)-\left({{\omega }_{1}+\gamma }^{X}+\mu \right){A}^{X}$$

10 $$\frac{d{A}_{R}^{X}}{dt}={\sigma }^{X}{\beta }_{R}^{X}{I}_{\text{total}}^{X}\sum_{Y\ne X}{R}^{Y}-\left({\gamma }^{X}+\mu \right){A}_{R}^{X}$$

The model assumes that an individuated infected by one variant cannot be infected by a second variant. Infected individuals recover and enter class **R**^**X**^ at a rate of γ^X^. Recovered individuals are entirely protected from the variant that they were infected with, but may be infected by a different variant at rate β^Y^_R_, where Y ≠ X, and β^Y^_R_ < β^Y^ to represent some degree of protection. Infected recovered individuals are denoted **I**^**X**^_**R**_, which like I^X^_V_ are associated with a better survival rate (μ^X^_R_ < μ^X^). Recovered individuals lose their partial immunity and enter S_VR_ at a rate of η_R_. Four recovered classes are represented, one for each virus type (R^W^, R^A^, R^D^) and also **R**^**F**^ who has recovered from two infections. For simplicity, we assume that R^F^ is fully immune to all three viruses, until they lose their immunity and become fully susceptible.

11 $$\frac{d{R}^{X}}{dt}={\gamma }^{X}\left({I}^{X}+{I}_{V}^{X}+{A}^{X}\right)-{R}^{X}\left(\sum_{Y\ne X}{\beta }_{R}^{Y}{I}_{\text{total}}^{Y}\right)-\left({\eta }_{R}+\mu \right){R}^{X}$$

12 $$\frac{d{R}^{F}}{dt}=\sum_{X=W,A,D}{\gamma }^{X}\left({I}_{R}^{X}+{A}_{R}^{X}\right)-\left({\eta }_{R}+\mu \right){R}^{F}$$

### Model parameters

The SV^2^(AIR)^3^ model involves parameters that describe the clinical characteristics of SARS-CoV-2 VOC, which were estimated from published studies. Other parameters describe the demographics and social behaviors of the Ontario population, which were estimated from published provincial statistics. Parameters were then fine-tuned manually so that model predictions approximate the published number of new infections and deaths (Ontario COVID-19 Science Advisory Table). Baseline parameter values are shown in Table S1.

### Simulating NPI

As the number of COVID-19 rose, on March 23 2020 the Ontario government began a number of public health safety measures, which varied in severity over time, including stay-at-home orders, workplace safety measures, restrictions on public events and social gatherings, temporary shutdown of selected businesses, and mandatory mask wearing. These NPI reduce the transmissivity of COVID-19, and are represented by scaling all β^X^’s and β^X^_V,M_’s simultaneously by a NPI severity index λ, where 0 ≤ λ ≤ 1. The time-varying function λ is chosen in part based on Ontario COVID-19 lockdown timeline and Google mobility data, and in part to fit the predicted new case numbers against provincial data available prior to July 2021; the values of λ for 2020–2021 are shown in Fig. S1.

## Supplementary Information

Supplementary Information.

## Data Availability

The computer code produced in this study is available in https://github.com/MehrshadSD/.
