# Supplementary material for: Understanding the dynamics of SARS-CoV-2 variants of concern in Ontario, Canada: a modeling study
Source: Sci Rep. 2022 Feb 8;12:2114. doi: 10.1038/s41598-022-06159-x (PMC8826311; doi:10.1038/s41598-022-06159-x)
Supplement: Supplementary file 1 — Supplementary Information. [file 41598_2022_6159_MOESM1_ESM.docx]

**Table S1**. Model parameters. Infection rates are given per individual per day; other rates are given per day.

^♣^2018-2019 death rate in Ontario, Statistics Canada.

^♦^Taken to be 70% higher than actual number of deaths / cases (July 2021, Statistics Canada) per day, since only infected susceptible die at this rate.

| Symbol | Description | Values |
| --- | --- | --- |
| α^W^, α^A^, α^D^ | Ratio of infectivity of asymptomatic and symptomatic individuals | 3.0 [1] |
| β^W^ | Rate of symptomatic infections of susceptibles by wild type | 3.3E-9 |
| β^A^ | Rate of symptomatic infections of susceptibles by Alpha | 5.5E-9 [2] |
| β^D^ | Rate of symptomatic infections of susceptibles by Delta | 7.6E-9 [2] |
| β^W^_V1,PZ_ | Rate of symptomatic infections of individuals vaccinated with one Pfizer dose by wild type | 0.2×β^W^ [3, 4] |
| β^W^_V2,PZ_ | Rate of symptomatic infections of individuals vaccinated with both Pfizer doses by wild type | 0.05×β^W^ [4] |
| β^A^_V1,PZ_ | Rate of symptomatic infections of individuals vaccinated with one Pfizer dose by Alpha | 0.5×β^A^ [5] |
| β^A^_V2,PZ_ | Rate of symptomatic infections of individuals vaccinated with both Pfizer doses by Alpha | 0.07×β^A^ [5] |
| β^D^_V1,PZ_ | Rate of symptomatic infections of individuals vaccinated with one Pfizer dose by Delta | 0.67×β^D^ [6] |
| β^D^_V2,PZ_ | Rate of symptomatic infections of individuals vaccinated with both Pfizer doses by Delta | 0.12×β^D^ [6] |
| β^W^_V1,AZ_ | Rate of symptomatic infections of individuals vaccinated with one AstraZeneca dose by wild type | 0.2×β^W^ [4] |
| β^W^_V2,AZ_ | Rate of symptomatic infections of individuals vaccinated with both AstraZeneca doses by wild type | 0.05×β^W^ [4] |
| β^A^_V1,AZ_ | Rate of symptomatic infections of individuals vaccinated with one AstraZeneca dose by Alpha | 0.5×β^A^ [7] |
| β^A^_V2,AZ_ | Rate of symptomatic infections of individuals vaccinated with both AstraZeneca doses by Alpha | 0.34×β^A [7]^ |
| β^D^_V1,AZ_ | Rate of symptomatic infections of individuals vaccinated with one AstraZeneca dose by Delta | 0.67×β^D^ [6] |
| β^D^_V2,AZ_ | Rate of symptomatic infections of individuals vaccinated with both AstraZeneca doses by Delta | 0.4×β^D^ [6] |
| β^X^_R_ | Rate of symptomatic infection of individual recovered from a different variant | 0.05×β^X^ |
| γ^W^, γ^A^, γ^D^ | Recovery rate | 1/28 [8] |
| ε_PZ_ | Fraction of Pfizer vaccines | 0.95 |
| η_V1_ | Rate of individuals with one vaccine dose losing immunity | 1/365 |
| η_V2_ | Rate of individuals with both vaccine doses losing immunity | 1/(4×365) |
| η_R_ | Rate of recovered individuals losing immunity | 1/(4×365) |
| μ | Natural death rate | 298.7^♣^ |
| μ^W^ | Death rate from wild-type infections | 0.001^♦^ |
| μ^A^ | Death rate from Alpha infections | 1.6×μ^W^ |
| μ^D^ | Death rate from Delta infections | 1.8×μ^W^ |
| μ^W^_V_, μ^A^_V_, μ^D^_V_ | Death rate of infected vaccinated individuals | 0.15×(μ^W^, μ^A^, μ^D^) |
| σ^W^, σ^A^, σ^D^ | Fraction of asymptomatic infections | 0.5 [9] |
| σ^W^_V_, σ^A^_V_, σ^D^_V_ | Fraction of asymptomatic infections among vaccinated individuals | 0.85 |
| σ^W^_R_, σ^A^_R_, σ^D^_R_ | Fraction of asymptomatic infections among recovered individuals | 0.85 |

**Vaccination rates**. Vaccination rates follow Ontario timeline. Vaccination is assumed to begin on t_vac_^0^ = December 14 2020. Vaccination rate increases linearly until it peaks at the end of June (t_vac_^1^ = June 30 2021). Prior to the end of May (t_vac_^2^ = May 31 2021), dosing interval is Δt^0^ = 16 weeks. Afterwards, dosing interval is Δt^PZ^ = 4 weeks for Pfizer and Δt^AZ^ = 8 weeks for AstraZeneca.

$$\omega_{1}(t)=\left\{ \begin{aligned} 0, t<t_{\text{vac}}^{0} \\ r_{0}\frac{t-t_{\text{vac}}^{0}}{t_{\text{vac}}^{1}-t_{\text{vac}}^{0}}, t_{\text{vac}}^{0}<t<t_{\text{vac}}^{1} \\ \left( r_{0}-r_{\text{ss}} \right)\text{exp}\left( -0.03\left( t-t_{\text{vac}}^{1} \right) \right)+r_{\text{ss}}, t_{\text{vac}}^{1}<t \end{aligned} \right.$$

$\omega_{2}^{M}(t)=\left\{ \begin{aligned} 0, t<t_{\text{vac}}^{0} \\ \frac{1}{\Delta t^{0}}, t_{\text{vac}}^{0}<t<t_{\text{vac}}^{2} \\ \frac{1}{\Delta t^{M}}, t_{\text{vac}}^{2}<t \end{aligned} \right.$, M = PZ, AZ

where r_0_ and r_ss_ are taken to be 1.1% and 0.05% of the population per day. These parameters are chosen so that at the end of July 2021, 71% of the population have received at least one dose and 57% are fully vaccinated, consistent with Ontario statistics (COVID19Tracker.ca).

**Non-pharmaceutical interventions (NPI)**. The NPI severity index (λ, shown in Fig. S1) is chosen in part based on Ontario COVID-19 lockdown timeline and Google mobility data, and in part to fit the predicted new case numbers against provincial data. Effective disease infectivity is obtained by scaling all infectivity parameters (β^X^’s and β^X^_V,M_’s) simultaneously.


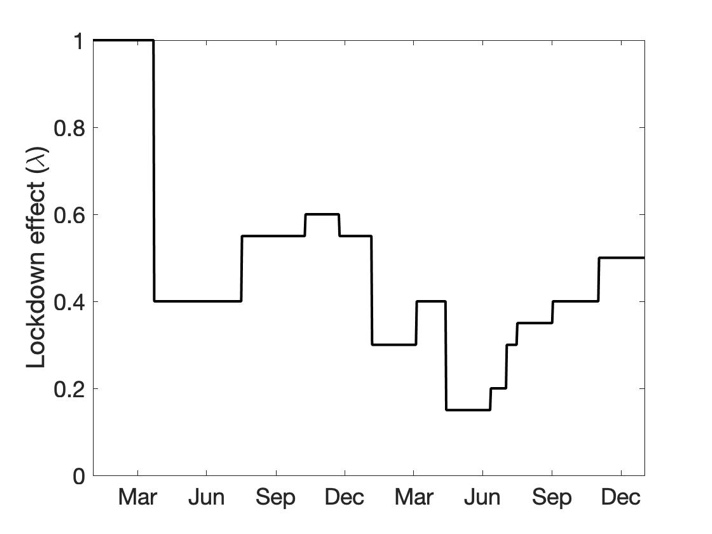


**Figure S1**. NPI index (λ) between January 1 2020 and December 31 2021.

References

1. Li, Y., et al., *Asymptomatic and symptomatic patients with non-severe coronavirus disease (COVID-19) have similar clinical features and virological courses: a retrospective single center study.* Frontiers in microbiology, 2020. **11**: p. 1570.

2. *Updates on COVID-19 Variants of Concern*. 2021, National Collaborating Ccentre for Infectious Diseases.

3. Thompson, M.G., et al., *Interim estimates of vaccine effectiveness of BNT162b2 and mRNA-1273 COVID-19 vaccines in preventing SARS-CoV-2 infection among health care personnel, first responders, and other essential and frontline workers—eight US locations, December 2020–March 2021.* Morbidity and Mortality Weekly Report, 2021. **70**(13): p. 495.

4. Bernal, J.L., et al., *Effectiveness of the Pfizer-BioNTech and Oxford-AstraZeneca vaccines on covid-19 related symptoms, hospital admissions, and mortality in older adults in England: test negative case-control study.* bmj, 2021. **373**.

5. Hall, V.J., et al., *Effectiveness of BNT162b2 mRNA vaccine against infection and COVID-19 vaccine coverage in healthcare workers in England, multicentre prospective cohort study (the SIREN Study).* 2021.

6. (WHO), W.H.O., *COVID-19 weekly epidemiological update*. 2021.

7. Emary, K.R., et al., *Efficacy of ChAdOx1 nCoV-19 (AZD1222) vaccine against SARS-CoV-2 variant of concern 202012/01 (B. 1.1. 7): an exploratory analysis of a randomised controlled trial.* The Lancet, 2021. **397**(10282): p. 1351-1362.

8. Barman, M.P., et al., *COVID-19 pandemic and its recovery time of patients in India: A pilot study.* Diabetes & Metabolic Syndrome: Clinical Research & Reviews, 2020. **14**(5): p. 1205-1211.

9. Oran, D.P. and E.J. Topol, *The proportion of SARS-CoV-2 infections that are asymptomatic: a systematic review.* Annals of internal medicine, 2021. **174**(5): p. 655-662.
